# Cytokine Adsorber Use during DCD Heart Perfusion Counteracts Coronary Microvascular Dysfunction

**DOI:** 10.3390/antiox11112280

**Published:** 2022-11-17

**Authors:** Lars Saemann, Fabio Hoorn, Adrian-Iustin Georgevici, Sabine Pohl, Sevil Korkmaz-Icöz, Gábor Veres, Yuxing Guo, Matthias Karck, Andreas Simm, Folker Wenzel, Gábor Szabó

**Affiliations:** 1 Department of Cardiac Surgery, University Hospital Halle, University of Halle, Ernst Grube Straße 40, 06120 Halle, Germany; 2 Department of Cardiac Surgery, Heidelberg University Hospital, 69120 Heidelberg, Germany; 3 Faculty Medical and Life Sciences, Furtwangen University, 78054 Villingen-Schwenningen, Germany; 4 Department of Anaesthesiology, St. Josef Hospital, Ruhr-University Bochum, 44791 Bochum, Germany

**Keywords:** heart transplantation, microvascular dysfunction, oxidative stress, donation after circulatory death, machine perfusion, cytokine adsorption, hemoadsorption, cytokines

## Abstract

Microvascular dysfunction (MVD) in cardiac allografts is associated with an impaired endothelial function in the coronary microvasculature. Ischemia/reperfusion injury (IRI) deteriorates endothelial function. Hearts donated after circulatory death (DCD) are exposed to warm ischemia before initiating ex vivo blood perfusion (BP). The impact of cytokine adsorption during BP to prevent MVD in DCD hearts is unknown. In a porcine DCD model, we assessed the microvascular function of hearts after BP with (DCD-BP^CytoS^, *n* = 5) or without (DCD-BP, *n* = 5) cytokine adsorption (CytoSorb^®^). Microvascular autoregulation was assessed by increasing the coronary perfusion pressure, while myocardial microcirculation was measured by Laser-Doppler-Perfusion (LDP). We analyzed the immunoreactivity of arteriolar oxidative stress markers nitrotyrosine and 4-hydroxy-2-nonenal (HNE), endothelial injury indicating cell adhesion molecules CD54, CD106 and CD31, and eNOS. We profiled the concentration of 13 cytokines in the perfusate. The expression of 84 genes was determined and analyzed using machine learning and decision trees. Non-DCD hearts served as a control for the gene expression analysis. Compared to DCD-BP, relative LDP was improved in the DCD-BP^CytoS^ group (1.51 ± 0.17 vs. 1.08 ± 0.17). Several pro- and anti-inflammatory cytokines were reduced in the DCD-BP^CytoS^ group. The expression of eNOS significantly increased, and the expression of nitrotyrosine, HNE, CD54, CD106, and CD31, markers of endothelial injury, majorly decreased in the DCD-BP^CytoS^ group. Three genes allowed exact differentiation between groups; regulation of HIF1A enabled differentiation between perfusion (DCD-BP, DCD-BP^CytoS^) and non-perfusion groups. CAV1 allowed differentiation between BP and BP^CytoS^. The use of a cytokine adsorption device during BP counteracts preload-dependent MVD and preserves the microvascular endothelium by preventing oxidative stress and IRI of coronary arterioles of DCD hearts.

## 1. Introduction

Coronary microvascular dysfunction (CMVD {XE “MVD” \t “Coronary Microvascular Dysfunction”}) in cardiac allografts is associated with a higher likelihood of death, graft failure, or allograft vasculopathy after heart transplantation [1]. CMVD is primarily characterized by impaired endothelial function in the myocardial microvasculature [2]. Coronary endothelial damage in cardiac allografts initiates a diversity of complex pathophysiological processes leading to the development of cardiac allograft vasculopathy, which is a common reason for long-term graft failure [3]. Prolonged ischemia and ischemia/reperfusion injury (IRI{XE “IRI” \t “ischemia/reperfusion injury”}) promote endothelial damage in donor hearts. In turn, IRI injury of the coronary endothelium is associated with oxidative stress, the expression of proinflammatory mediators, and platelet adherence—well-known mechanisms that lead to further damage of the coronary vasculature and increased CMVD [4,5].

Some transplant centers have established heart transplantation programs with hearts harvested from donors after circulatory death (DCD{XE “DCD” \t “Donation after Circulatory Death”}) [6]. DCD hearts must withstand a warm ischemic period followed by reperfusion during transportation by normothermic blood perfusion (BP{XE “BP” \t “Blood Perfusion”}) with blood collected from the deceased donor [6]. Both brain-dead and DCD donors increase proinflammatory cytokine levels before organ harvesting and blood collection. They could potentially exacerbate vascular damage during BP of donor hearts with the blood of the respective donor.

Considering this, we investigated the effect of cytokine adsorption during blood perfusion of DCD hearts on CMVD, oxidative stress, and IRI of the coronary microvascular endothelium. We also determined key regulatory gene expression using decision trees.

## 2. Materials and Methods

### 2.1. Animals and Anesthesia

The investigations were reviewed and approved (35-9185.81/G-150/19) by the appropriate institutional Ethical Committee for Animal Experimentation. The animals received humane care. We sedated healthy pigs (40–50 kg bodyweight) with intramuscular injection of ketamine (22.5 mg/kg; Bremer Pharma, Warburg, Germany) and midazolam (0.375 mg/kg; Hameln pharma plus, Hameln, Germany). Anesthesia was maintained intravenously with pentobarbital-sodium (15 mg/kg/h; Boehringer Ingelheim Vetmedicia, Ingelheim, Germany). 

### 2.2. Donation after Circulatory Death Model and Harvesting of Hearts

According to a previously published model, we induced circulatory death through the termination of mechanical ventilation [7]. Within the subsequent period of 30 min, we collected blood and harvested the heart. After a total warm ischemic period of 30 min, we flushed the DCD hearts with 2 L of cold (4 °C) Custodiol solution (Köhler Chemie GmbH, Bensheim, Germany), followed by mounting on the perfusion system.

### 2.3. Blood Perfusion and Study Groups

**Figure 1:**
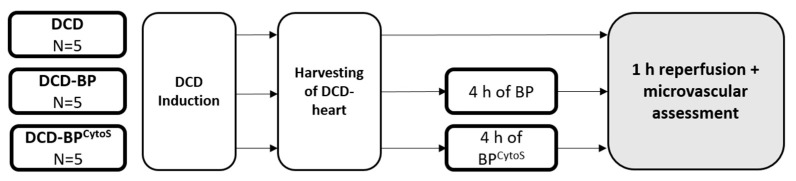
Workflow. BP: blood perfusion. DCD: donation after circulatory death. BP^CytoS^: blood perfusion with an integrated cytokine adsorption device.

DCD hearts were maintained for four hours by normothermic blood perfusion through the ascending aorta with (DCD-BP^CytoS^, *n* = 5) or without (DCD-BP, *n* = 5) a cytokine adsorber (CytoSorb^®^, Cytosorbents Europe, Berlin, Germany). Additionally, we performed two control groups for the molecular biological analysis: DCD hearts and native control hearts, without 4 h of maintenance perfusion. Finally, the hearts of all groups underwent 60 min of perfusion with fresh blood to mimic the reperfusion effects after transplantation (Figure 1).

### 2.4. Microvascular Functional Assessment

We determined the regulation of coronary macro- and microvascular blood flow by measuring total coronary flow and myocardial microcirculation while the coronary perfusion pressure (CPP{XE “CPP” \t “Coronary Perfusion Pressure”}) was adjusted stepwise between 20 and 100 mmHg [8]. We measured myocardial microcirculation through laser Doppler perfusion monitoring [9]. Myocardial microcirculation is proportional to the laser Doppler perfusion (LDP{XE “LDP” \t “Laser-Doppler-Perfusion”}) signal. We inserted a laser Doppler needle probe into the left ventricular anterior wall. Due to the dimensionless properties of LDP, the signal needs to be related to a baseline measurement, leading to relative LDP (RLDP{XE “RLDP” \t “Relative LDP”}). A baseline measurement was performed with a CPP of 20 mmHg and an LVV of 5 mL. We also determined the left-ventricular myocardial workload at different preload volumes and, meanwhile, measured LDP.

### 2.5. Cytokine Profiling

We profiled the perfusate concentration of 13 cytokines from serum samples using a Porcine Cytokine/Chemokine Magnetic Bead Panel (Milliplex^®^ Map Kit, EMD Millipore Corporation, Merck, Darmstadt, Germany) and a Bio-Plex 200 (Biorad, Feldkirchen, Germany) according to the manufacturer’s instructions.

### 2.6. Immunohistochemical Staining

After the functional assessment, samples of LV myocardial tissue were frozen in liquid nitrogen and stored at −80 °C or in a paraformaldehyde solution. Immunohistochemical staining of endothelial nitric oxide synthase (eNOS; 1:100; Cell signaling technology, Danvers, MA, USA{XE “eNOS” \t “Endothelial Nitric Oxide Synthase”}), CD54 (1:100; Novusbio, Wiesbaden, Germany{XE “ICAM” \t “Intracellular Cell Adhesion Molecule-1”}), CD106 (1:1000; Santa Cruz Biotechnology, Heidelberg, Germany{XE “VCAM” \t “Vascular Cell Adhesion Molecule-1”}), CD31 (1:1000; Santa Cruz Biotechnology, Heidelberg, Germany{XE “PECAM-1” \t “Platelet Endothelial Cell Adhesion Molecule-1”}{ XE “CD-31” \t “Cluster of Differentiation-31”}), markers of endothelial injury, nitrotyrosine (1:200, hm5101, Hycult Biotech, Wayne, PA, USA), 4-hydroxy-2-nonenal (HNE{XE “HNE” \t “4-Hydroxy-2-Nonenal”}; 1:2500, mab3249, R&D System, Minneapolis, MN, USA), and markers of oxidative stress was performed according to the manufacturer’s instructions.

### 2.7. Gene Expression Analysis

The expression of 84 genes was analyzed using the RT^2^ PCR array (Qiagen, Hilden, Germany) as described elsewhere [9].

### 2.8. Statistical Analysis and Machine Learning Algorithm

Statistical analysis was performed using IBM SPSS Statistics for Windows (Version 25.0, IBM Corp. Armonk, NY, USA). Results are expressed as mean ± standard error. We tested for homogeneity of variances using the Levene test. Data were analyzed using a one-way analysis of variance for multiple comparisons with Tukey adjustment of *p*-values in case of variance homogeneity, and Games–Howell adjustment in case of variance inhomogeneity. For LDP analysis, a two-tailed unpaired classical t-test was applied in case of variance homogeneity and a Welch t-test in case of variance inhomogeneity. A value of *p* < 0.05 was considered statistically significant in all analyses, and a value of *p* < 0.001 was considered statistically highly significant.

We compared gene expression between groups using a two-tailed unpaired t-test with Benjamini–Hochberg corrected *p*-values. A principal component analysis was made for genes preselected using the Borruta algorithm, a machine learning algorithm based on a permutated random forest [10]. We also constructed a decision tree confusion matrix to identify key regulated genes, which allows differentiation between groups.

## 3. Results

### 3.1. Microvascular Function

**Figure 2:**
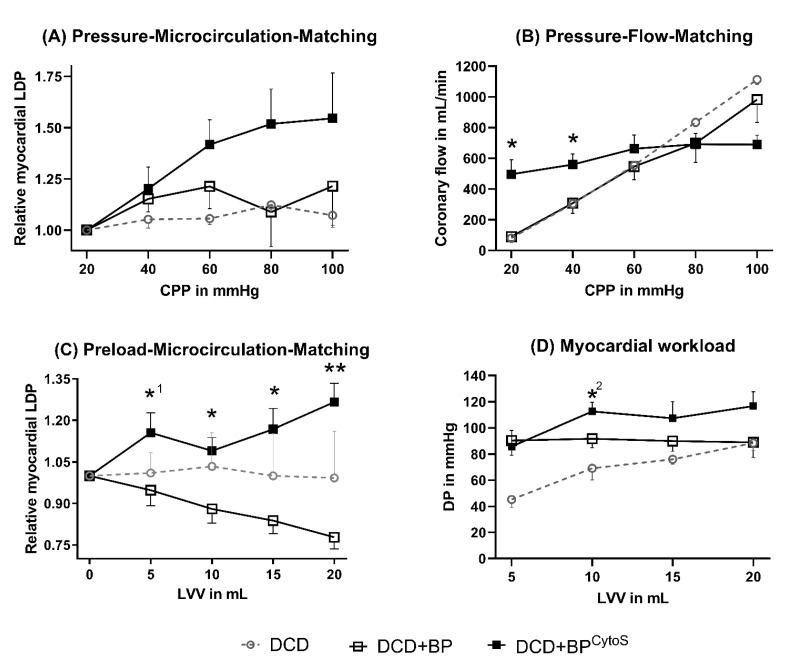
Microvascular function. (**A**) Pressure-Microcirculation-Matching. (**B**) Pressure-Flow-Matchig. (**C**) Preload-Microcirculation-Matching. (**D**) Myocardial workload * *p* < 0.05, *^1^ *p* = 0.053, *^2^ *p* = 0.067 and ** *p* < 0.001 compared to DCD-BP. LDP in DCD hearts is only shown as a reference. BP: blood perfusion. BP^CytoS^: blood perfusion with an integrated cytokine adsorption device. DCD: donation after circulatory death. DP: developed pressure. LDP: laser Doppler perfusion. LV: left-ventricular.

In DCD-BP, RLDP was decreased compared to DCD-BP^CytoS^, with a major difference at high CPP conditions (Figure 2A). At the same time, coronary flow significantly differed in DCD-BP compared to DCD-BP^CytoS^ in dependence on CPP (Figure 2B). While coronary flow and CPP showed almost a linear relationship in DCD-BP, the flow curve showed a flattened, asymptotic profile in DCD-BP^CytoS^. Even at a low CPP of 20 and 40 mmHg, coronary flow remained high and was significantly increased compared to DCD-BP. During preload-microcirculation matching, RLDP was highly significantly improved in DCD-BP^CytoS^ compared to DCD-BP. The myocardial workload was also improved in DCD-BP^CytoS^.

### 3.2. Perfusion Pressure

**Figure 3:**
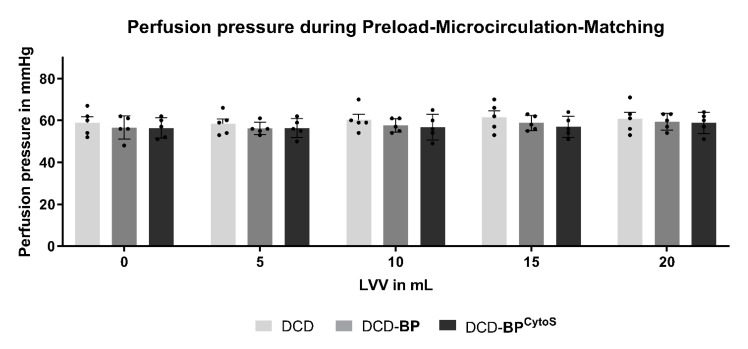
Perfusion pressure. Light grey: DCD. Grey: DCD-BP. Dark grey: DCD-BP^CytoS^. LV: left ventricular. MP: machine perfusion. BP: blood perfusion. DCD: donation after circulatory death. BP^CytoS^: blood perfusion with an integrated cytokine adsorption device.

The CPP was analyzed in parallel to exclude a possible impact of the CPP on the RLDP results. The CPP was comparable between all groups at all LVVs during microvascular assessment (Figure 3).

### 3.3. Cytokine Profile

**Figure 4:**
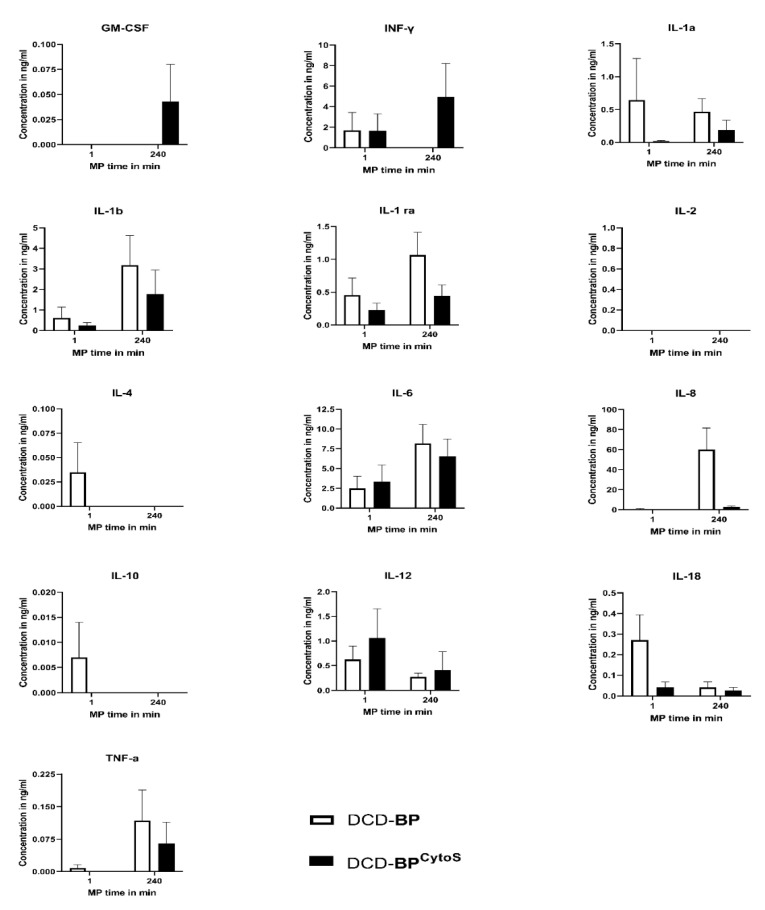
Cytokine profile. IL: interleukine. INF: interferone. TNF: tumor necrosis factor. MP: machine perfusion. BP: blood perfusion. DCD: donation after circulatory death. BP^CytoS^: blood perfusion with an integrated cytokine adsorption device.

The microvascular function can be influenced by inflammation. Therefore, we determined pro- and anti-inflammatory cytokines using a porcine cytokine/chemokine multiplex array system. The cytokine profile in the perfusate is shown in Figure 4. The concentration of several cytokines was decreased in DCD-BP^CytoS^, such as interleukin (IL{XE “IL” \t “Interleukine”})-1a, -1b, -1ra, -8, -18, and TNF-α. IL-4 and -10 could only be determined in DCD-BP at the beginning of perfusion in very low concentrations. GM-CSF and INF-γ were only detected in DCD-BP^CytoS^ after 240 min of perfusion. IL-6 and -12 did not significantly differ between the groups. We could not detect IL-2 in either of the groups. The concentration of cytokines increased by perfusion time after 4 h with a marked increase, especially in DCD-BP.

### 3.4. Oxidative Stress and Endothelial Injury

**Figure 5:**
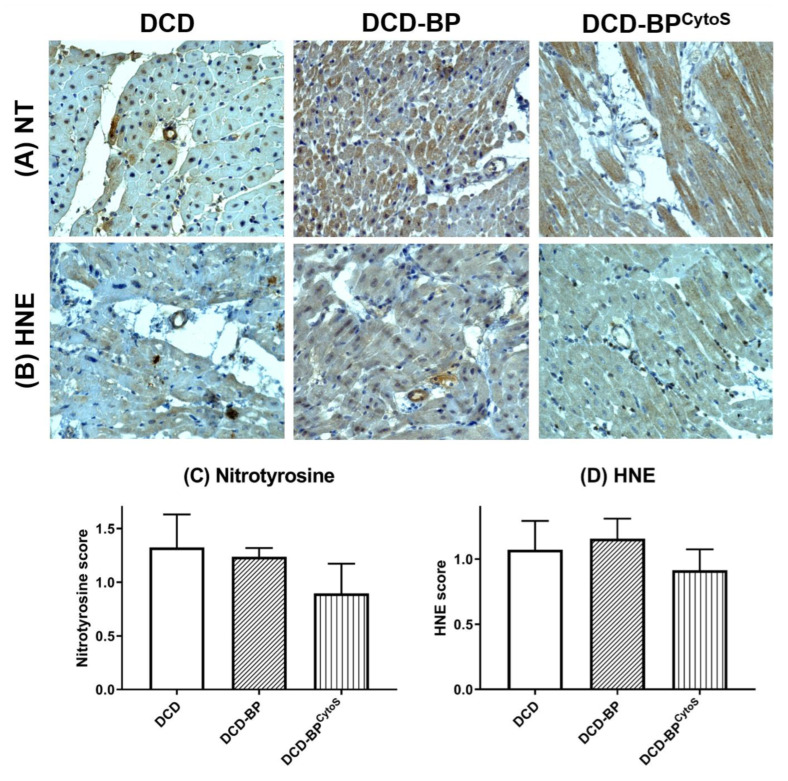
Representative photomicrographs of immunohistochemical staining of endothelial oxidative stress (**A**,**B**). Semiquantitative analysis of arteriolar immunoreactivity (**C**,**D**). BP: blood perfusion. CD: cluster of differentiation. DCD: donation after circulatory death. BP^CytoS^: blood perfusion with an integrated cytokine adsorption device. NT: nitrotyrosine. HNE: hydrxynonenal.

**Figure 6:**
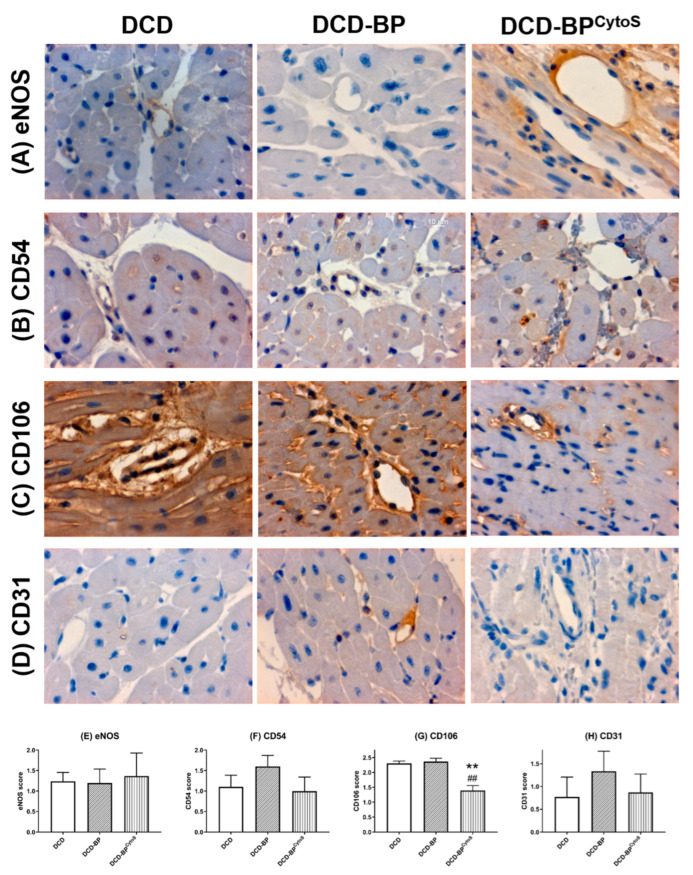
Representative photomicrographs of immunohistochemical staining of injury markers (**A**–**D**). Semiquantitative analysis of arteriolar immunoreactivity (**E**–**H**). ** *p* < 0.001 compared to DCD. ^##^ *p* < 0.001 compared to DCD-BP. BP: blood perfusion. CD: cluster of differentiation. DCD: donation after circulatory death. BP^CytoS^: blood perfusion with an integrated cytokine adsorption device. eNOS: endothelial nitric oxide synthase.

Endothelial oxidative stress and function/injury parameters were analyzed in arterioles of the left ventricular myocardium by immunohistochemical analysis. Nitrotysine and HNE were expressed lower in DCD-BP^CytoS^ compared to DCD-BP (Figure 5). Blood perfusion without cytokine adsorption resulted in an eNOS score comparable to the DCD group (Figure 6). The expression of CD54, CD106, and CD31 decreased in DCD-BP^CytoS^ (Figure 6).

### 3.5. Gene Expression

**Figure 7:**
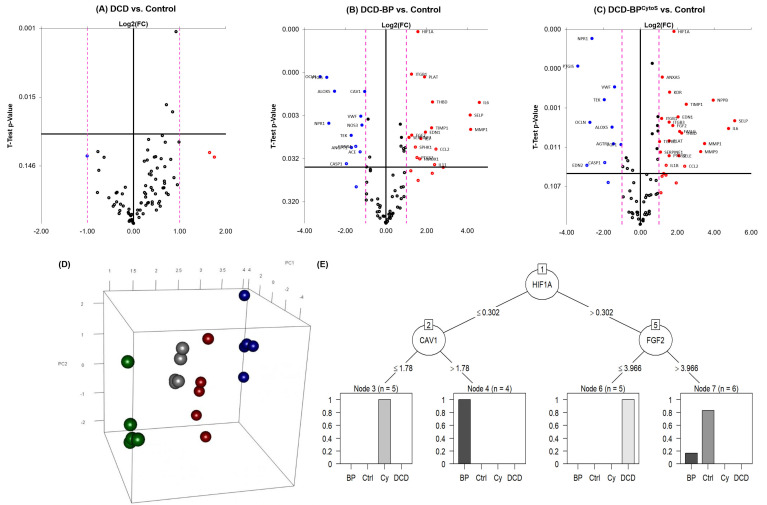
(**A**) Volcano plots for regulated genes of the group DCD, (**B**) DCD-BP, and (**C**) DCD-BP^CytoS^ compared to Control. (**D**) Principal component analysis on the Borruta preselected variables, (**E**) Decision tree to show key regulated genes, which allow differentiation between groups. BP: blood perfusion. BP^CytoS^ or Cy: blood perfusion with an integrated cytokine adsorption device. Crtl: control. DCD: donation after circulatory death.

Myocardial samples from the hearts of the DCD group did not show significantly up- or downregulated genes compared to the non-DCD control group (Figure 7A). Instead, hearts from the perfusion groups showed an extensive profile of significantly regulated genes (Figure 7B,C). The principal component analysis showed clear separation of groups based on the Borruta preselected variables (Figure 7D). We identified three genes that allow differentiation between experimental groups: Regulation of hypoxia-inducible factor 1a (HIF1A{XE “HIF1A” \t “Hypoxia Inducible Factor 1a”}) allows differentiation between perfusion groups (DCD-BP and DCD-BP^CytoS^) and non-perfusion groups. By regulation of caveolin 1 (CAV1{XE “CAV1” \t “Caveolin 1”}), BP hearts can be separated from those that received cytokine adsorption therapy. Fibroblast growth factor 2 (FGF2{XE “FGF2” \t “Fibroblast growth factor 2”}) allows differentiation between the DCD and the control group.

## 4. Discussion

We demonstrated (1) a decreased CMVD, (2) reduced cytokine concentrations, (3) reduced level of oxidative stress and an alleviated microvascular endothelial IRI, and (4) key regulated genes in porcine DCD hearts after cytokine adsorption during normothermic ex vivo blood perfusion.

### 4.1. Microvascular Functional Effects

Different methods can assess coronary microvasculature. First, the assessment aims to characterize either the morphohistological or the functional status of microcirculatory vessels, predominantly arterioles [2]. In clinical practice, the coronary flow reserve is the most common method to evaluate coronary microvascular function [1]. Furthermore, the functional status is assessed using pharmacological stimulation of vasodilative mechanisms. Nevertheless, this method does not allow the evaluation of the autoregulation of coronary microcirculation. Instead, variation of the CPP can assess the regulative function of coronary microcirculatory vessels [8]. In healthy hearts, the coronary flow is regulated predominantly by arterioles that dilate or contract to keep the coronary circulation constant—a phenomenon known as the Bayliss effect [11]. Under physiological conditions, the flow–pressure relationship is linear in small pressure ranges due to maximally dilated vessels and shows a flattened profile at higher pressures. In the present study, the relationship of CPP and coronary flow was steep and linear in DCD-BP hearts over all CPPs. In DCD-BP^CytoS^ hearts, a high flow was maintained at low CPP, and the flow curve was linear only at low and then flattened at high CPP ranges. Therefore, DCD-BP^CytoS^ hearts were able to compensate for low CPPs well. The significant upregulation of the coronary microcirculation by CPP elevations in the low CPP range in DCD-BP^CytoS^ and the minor upregulation in the DCD-BP group indicate that treatment of DCD hearts with CytoSorb^®^ leads to the ability of autoregulative compensation of low CPP ranges in the coronary circulation. Instead, the minor microcirculatory change, despite a high coronary flow at a high CPP range in DCD-BP^CytoS^, indicates that without CytoSorb^®^ use, most of the coronary flow might pass arterio-venous anastomoses when the CPP has been increased. In the case of blood perfusion within the organ care system, optimizing the perfusion by elevating the CPP and/or increasing the infusion of the maintenance solution has to be carefully considered because the actual effective tissue perfusion, which is the coronary microcirculation, might only change slightly. This could be a potential reason for the well-known cases of elevating lactate concentrations during heart perfusion [12].

Myocardial microcirculation is also upregulated in an autoregulative manner by an increased LV preload, as Kjekshus et al. demonstrated in an experimental research project on dogs using labeled microspheres [13]. The present study confirms that preload-dependent microvascular flow regulation is impaired in porcine DCD hearts and in DCD hearts that underwent standard blood perfusion. Furthermore, this study shows that in DCD hearts that underwent BP using cytokine adsorption, the regulation of microvascular flow in dependence on LV preload is existent. Next to possible effects on the cardiomyocyte level, this reestablished regulation of microvascular flow and, therefore, improved myocardial supply could also be a reason for the increased myocardial workload in DCD-BP^CytoS^.

### 4.2. Cytokine Profile

We could show that CytoSorb^®^ modified the cytokine profile in the perfusate. This may have caused the microvascular functional improvement. Nevertheless, it has to be considered that CytoSorb^®^ is not selective and therefore does not only reduce proinflammatory mediators. The adsorption device presumably can bind a diversity of other molecules that might also affect microvascular function. Further work is required to characterize the binding properties not only for cytokines but also for proteins and metabolites, if possible, in an omics-based manner.

### 4.3. Oxidative Stress and Endothelial Injury

The vascular endothelium is of major importance for regulating blood flow in cardiac allografts [14]. Endothelial dysfunction induced by IRI has already been shown to reduce myocardial reperfusion due to vascular occlusion [15]. As demonstrated by the expression of cell adhesion molecules, endothelial IRI was increased without cytokine adsorption and could have been a driving factor for impaired microvascular function.

Oxidative stress commonly occurs during the reoxygenation of ischemic tissue and promotes endothelial injury and, consequently, endothelial dysfunction. Reactive oxygen species (ROS{ XE “ROS” \t “Reactive Oxygen Species” }) are key mediators of oxidative stress. A significant part of ROS is released from injured endothelial cells after reperfusion and contributes to an enhanced I/R injury. TNF-α, which was reduced in DCD-BP^CytoS^, induces ROS formation in the mitochondria of endothelial cells [16]. Furthermore, it was shown in pig coronary arterioles that TNF-α activates the enzyme xanthine oxidase, producing ROS [17]. ROS have also been shown to induce CD54, which was increased in DCD-BP but not in DCD-BP^CytoS^, in endothelial cells [18]. Both TNF-α and IL-1b can induce IL-8 [19]. Thus, next to the binding of IL-8 to the cytokine adsorber, a decreased induction of IL-8 might have been another reason for the major reduction of IL-8 in DCD-BP^CytoS^.

CD106 promotes the adhesion of leukocytes to endothelial cells and is, therefore, involved in inflammatory processes and endothelial activation [20]. This may be the reason for the decreased microvascular function in DCD-BP. Furthermore, the expression of CD106 can be stimulated by TNF-α and IL-1b [21]. Both cytokines, but especially IL-1b, were decreased in DCD-BP^CytoS^, which could be a potential explanation for the decreased expression of CD106 in the coronary arterioles in DCD-BP^CytoS^ hearts.

CD31 is a multifunctional molecule in vascular cell physiology. It plays a significant role in exacerbating the pathological effects of vascular IRI by promoting leukocyte infiltration and increasing platelet adherence and endothelial permeability [22]. It has been shown that the inactivation of CD31 by neutralizing antibodies reduces pathological effects of the adhesion molecule in response to proinflammatory stimuli or an environment of ischemia/reperfusion [22].

Nitric oxide is a potent vasodilator produced by eNOS and is released from endothelial cells in response to various physiological stimuli [23]. LV filling was shown to directly impact eNOS release [24]. CD31 also interacts with eNOS in endothelial cells [25,26]. The slightly increased eNOS expression in DCD-BP^CytoS^ suggests some additional endothelial protective effects of cytokine adsorption.

Furthermore, a reduced bioavailability of nitric oxide due to a reduced expression or activity of eNOS was shown to be associated with the impaired endothelial function of the coronary vasculature in the context of heart transplantation [14]. CD54 is involved in, and is stimulated by, inflammatory processes in the coronary vascular endothelium [27]. Furthermore, it has been identified as a potential predictor and marker of coronary allograft vasculopathy [28].

### 4.4. Gene Expression

Gene expression analysis was performed from left-ventricular tissue samples, which contained predominantly myocardium but also vascular tissue. Nevertheless, it is known that within a tissue compound, genes expressed from one type of cells, e.g., cardiomyocytes, also affect the cells of the surrounding tissue, e.g., endothelial cells and vascular smooth muscle cells. Conceivably, significantly regulated genes that were potentially expressed by the myocardium, if not even by arterioles themselves, would most likely also have had an effect on the arterioles. On the gene expression level, the perfusion of DCD hearts with blood leads to an upregulation of the plasminogen pathway, including plasminogen activator (PLAT{ XE “PLAT” \t “Plasminogen Activator” }) and plasminogen activator inhibitor 1 (SERPRINE1{ XE “SERPRINE1” \t “Plasminogen Activator Inhibitor 1” }), and plasminogen activator urokinase (PLAU{ XE “PLAU” \t “Plasminogen Activator Urokinase” }). Nitric oxide synthase 3 (NOS3{ XE “NOS3” \t “Nitric Oxide Synthase 3, eNOS”}, eNOS), which also showed a reduced immunoreactivity in the DCD-BP group, was downregulated in blood-perfused hearts without cytokine adsorption. Therefore, it is conceivable that eNOS could potentially stay reduced even longer than in a short-term manner. Furthermore, integrin beta 1 (ITGB1{ XE “ITGB1” \t “Integrin Beta 1” }), also upregulated, is involved in cytokinesis [29]. Three proinflammatory cytokines, interleukin (IL{ XE “IL” \t “Interleukin” })-6, -7, and -11, were also upregulated in DCD-BP compared to control. Overall, we found three key regulations of gene expression: HIF1A, CAV1, and FGF-2. HIF1A plays an essential role in adapting tissue to hypoxic conditions and is also known for its cardioprotective properties [30]. Furthermore, it is involved in angiogenesis and vascular remodeling during or in response to hypoxia [31]. Blood perfusion, whether with or without cytokine adsorption, facilitates a sufficient oxygen supply of the heart. Consequently, the reason for the differential HIF1a regulation between the perfusion and non-perfusion groups must be different. Presumably, cardioplegia delivery, which was performed in all groups, triggers HIF1a regulation, and during perfusion, this regulation could already be counter-regulated due to the 4 h of perfusion. CAV1, which was also identified by the decision tree as a key regulated gene, inhibits eNOS activity through direct binding and could therefore be a reason for the inferior microvascular function in DCD-BP compared to DCD-BP^CytoS^ [32].

### 4.5. Limitations

LDP monitoring is characterized by a high temporal resolution and therefore allows the detection of small changes in microvascular perfusion. Nevertheless, measurement circumstances can affect the LDP signal. Therefore, LDP measurements must follow a strict protocol, including a replicable technique applied by experienced investigators. The LDP signal is proportional to the local microcirculation in various validated tissues. However, validation experiments must be performed to determine the exact correlation between the LDP signal and absolute microcirculation in myocardial tissue.

## 5. Conclusions

Integration of CytoSorb^®^ into the machine perfusion system counteracts CMVD in a porcine model of DCD. Furthermore, CytoSorb^®^ use preserves the microvascular endothelium of DCD hearts and reduces oxidative stress and ischemia/reperfusion injury of coronary arterioles. In complex regulated gene expression, machine learning, notably the Borruta algorithm, provides an innovative way to identify relevant variables—in this case, genes. Our findings suggest that the use of CytoSorb^®^ in BP of DCD hearts could be beneficial in the clinical setting.

## Data Availability

All data is contained within the article.

## Abbreviations

**BP**	**blood perfusion**
**CAV1**	**caveolin 1**
**CD-31**	**cluster of differentiation-31**
**CPP**	**coronary perfusion pressure**
**DCD**	**donation after circulatory death**
**eNOS**	**endothelial nitric oxide synthase**
**FGF2**	**fibroblast growth factor 2**
**HIF1A**	**hypoxia inducible factor 1a**
**ICAM**	**intracellular cell adhesion molecule-1**
**IL**	**interleukin, interleukine**
**IRI**	**ischemia/reperfusion injury**
**ITGB1**	**integrin beta 1**
**LDP**	**laser-doppler-perfusion**
**CMVD**	**coronary microvascular dysfunction**
**NOS3**	**nitric oxide synthase 3, eNOS**
**PECAM-1**	**platelet endothelial cell adhesion molecule-1**
**PLAT**	**plasminogen activator**
**PLAU**	**plasminogen activator urokinase**
**RLDP**	**relative LDP**
**ROS**	**reactive oxygen species**
**SERPRINE1**	**plasminogen activator inhibitor 1**
**VCAM**	**vascular cell adhesion molecule-1**
